# Role of allopregnanolone-mediated γ-aminobutyric acid A receptor sensitivity in the pathogenesis of premenstrual dysphoric disorder: Toward precise targets for translational medicine and drug development

**DOI:** 10.3389/fpsyt.2023.1140796

**Published:** 2023-03-02

**Authors:** Qian Gao, Wei Sun, Yue-Rui Wang, Zi-Fa Li, Feng Zhao, Xi-Wen Geng, Kai-Yong Xu, Dan Chen, Kun Liu, Ying Xing, Wei Liu, Sheng Wei

**Affiliations:** ^1^Experimental Center, Shandong University of Traditional Chinese Medicine, Jinan, China; ^2^Key Laboratory of Traditional Chinese Medicine Classical Theory, Ministry of Education, Shandong University of Traditional Chinese Medicine, Jinan, China; ^3^Chinese Medicine and Brain Science Core Facility, Shandong University of Traditional Chinese Medicine, Jinan, China; ^4^Department of Encephalopathy, The Second Affiliated Hospital of Shandong University of Traditional Chinese Medicine, Jinan, China

**Keywords:** premenstrual dysphoric disorder, premenstrual syndrome, pathogenesis, allopregnanolone, γ-aminobutyric acid A receptor, subunit

## Abstract

Premenstrual dysphoric disorder (PMDD) can be conceptualized as a disorder of suboptimal sensitivity to neuroactive steroid hormones. Its core symptoms (emotional instability, irritability, depression, and anxiety) are related to the increase of stress sensitivity due to the fluctuation of hormone level in luteal phase of the menstrual cycle. In this review, we describe the emotional regulatory effect of allopregnanolone (ALLO), and summarize the relationship between ALLO and γ-aminobutyric acid A (GABA_A_) receptor subunits based on rodent experiments and clinical observations. A rapid decrease in ALLO reduces the sensitivity of GABA_A_ receptor, and reduces the chloride influx, hindered the inhibitory effect of GABAergic neurons on pyramidal neurons, and then increased the excitability of pyramidal neurons, resulting in PMDD-like behavior. Finally, we discuss in depth the treatment of PMDD with targeted GABA_A_ receptors, hoping to find a precise target for drug development and subsequent clinical application. In conclusion, PMDD pathophysiology is rooted in GABA_A_ receptor sensitivity changes caused by rapid changes in ALLO levels. Targeting GABA_A_ receptors may alleviate the occurrence of PMDD.

## 1. Introduction

Premenstrual syndrome (PMS) refers to a series of physical and emotional symptoms that women of childbearing age regularly experience before menstruation, such as anxiety, quick temper, excessive breast tenderness, increased or decreased appetite, nausea, vomiting, acne, low back pain, or fainting (1). Premenstrual dysphoric disorder (PMDD) is a debilitating subtype of PMS, and the typical symptoms of PMDD include emotional instability, irritability, depression, anxiety, decreased interest in daily activities, inattention, fatigue, appetite changes, sleep changes, feeling overwhelmed, and other emotional symptoms, with physical symptoms such as headache, edema, and breast pain (2, 3). The American Psychiatric Association published the diagnostic criteria for PMDD in the Diagnostic and Statistical Manual of Mental Disorders (DSM) (4). The essential features of premenstrual dysphoric disorder are the expression of mood lability, irritability, dysphoria, and anxiety symptoms that occur repeatedly during the premenstrual phase of the cycle and remit around the onset of menses or shortly thereafter. These symptoms may be accompanied by behavioral and physical symptoms. Symptoms must have occurred in most of the menstrual cycles during the past year and must have an adverse effect on work or social functioning (5). Symptoms must appear in the last week before menstruation begins, and begin to improve within a few days after menstruation begins. Given that the menstrual cycle lasts 4 weeks, few weeks after menstruation almost coincides with the week preceding menstruation in which PMS/PMDD symptoms manifest. At least five symptoms must appear, including a “core” symptom (obvious emotional instability, irritability, depression, or anxiety) and other potential symptoms, including decreased interest in daily activities, difficulty in focusing, insufficient energy, sleep or appetite change, feeling overwhelmed or out of control, and physical symptoms. The incidence rate of PMS is 30–40%, and the incidence rate of PMDD is 3–8% (6, 7). The rates of long-term and 12-month suicidal thoughts in patients with PMDD were 45.8 and 18.6%, respectively (8). It is particularly necessary to find a way to treat PMDD.

At present, the first-line drugs for treatment of PMS/PMDD are selective serotonin reuptake inhibitors (SSRIs), such as fluoxetine, sertraline, and paroxetine (9, 10). The effective response rate of PMS/PMDD to SSRIs is 60–90%, while that to placebo is 30–40% (11, 12). However, symptoms such as nausea, weakness, drowsiness, fatigue, decreased libido, and sweating are side effects of SSRIs. Because of the long half-life of SSRIs, these symptoms usually become more and more serious and last for several weeks (13). Because of these adverse side effects, there is poor patient compliance with SSRI treatment, and many PMDD patients choose to terminate the therapy. Therefore, it is urgent to find alternative or supplementary therapeutic methods or discover new treatment targets for PMDD.

In recent years, studies have found that the symptoms of PMDD are greatly affected by the fluctuation of neurosteroids during the menstrual cycle (14). It is well known that the progesterone content in normal animals will decrease sharply, and the estrogen in normal animals will remain at a stable low level, during the late period of estrus. At this stage, some animals have a strong sensitivity to the rapid decline of progesterone level in their bodies, usually showing obvious depression and PMDD-like behavior (15). Therefore, the rapid decrease of progesterone content in the luteal phase may be a trigger factor for female animals to be vulnerable to psychological stress. At the same time, the rapid decrease of progesterone content in the luteal phase is also a major feature of PMDD. Therefore, it is a method to treat PMDD by supplementing progesterone level to delay the rapid decline of progesterone. As a metabolite of progesterone, allopregnanolone (ALLO) has received therapeutic approval for postpartum depression (PPD) (16), which is similar to PMDD and also related to the abnormal changes of hormone in the key period of female reproductive physiological cycle. Further studies (17–19) show that ALLO is a positive allosteric modulator of the γ-aminobutyric acid A (GABA_A_) receptor. The therapeutic effect of ALLO supplementation against PMDD may be achieved by regulating the expression and function of GABA_A_ receptor. Therefore, this article will review the pathogenesis of PMDD caused by ALLO-mediated changes in GABA_A_ receptor sensitivity, with the aim of finding precise targets for drug development and subsequent clinical applications.

## 2. Effect of ALLO on emotional regulation

Neurosteroids are key molecules in the central nervous system that regulate neural function. Through the interaction with ion channel-coupled receptors, they can quickly change neural excitability. Neurosteroids are produced in the brain and peripheral nervous system, and also some endocrine glands (adrenal glands and gonads) in the body that produce steroidal compounds or steroidal substances with neural activity, including glucocorticoids, mineralocorticoids, progesterone, androgens, and estrogen (20). Neurosteroids are widely involved in human physiological and pathological processes. In the menstrual cycle and other physiological processes, the level of neurosteroids fluctuates, and mental diseases such as depression and anxiety are closely related to neurosteroids and their receptors (21).

**Figure 1:**
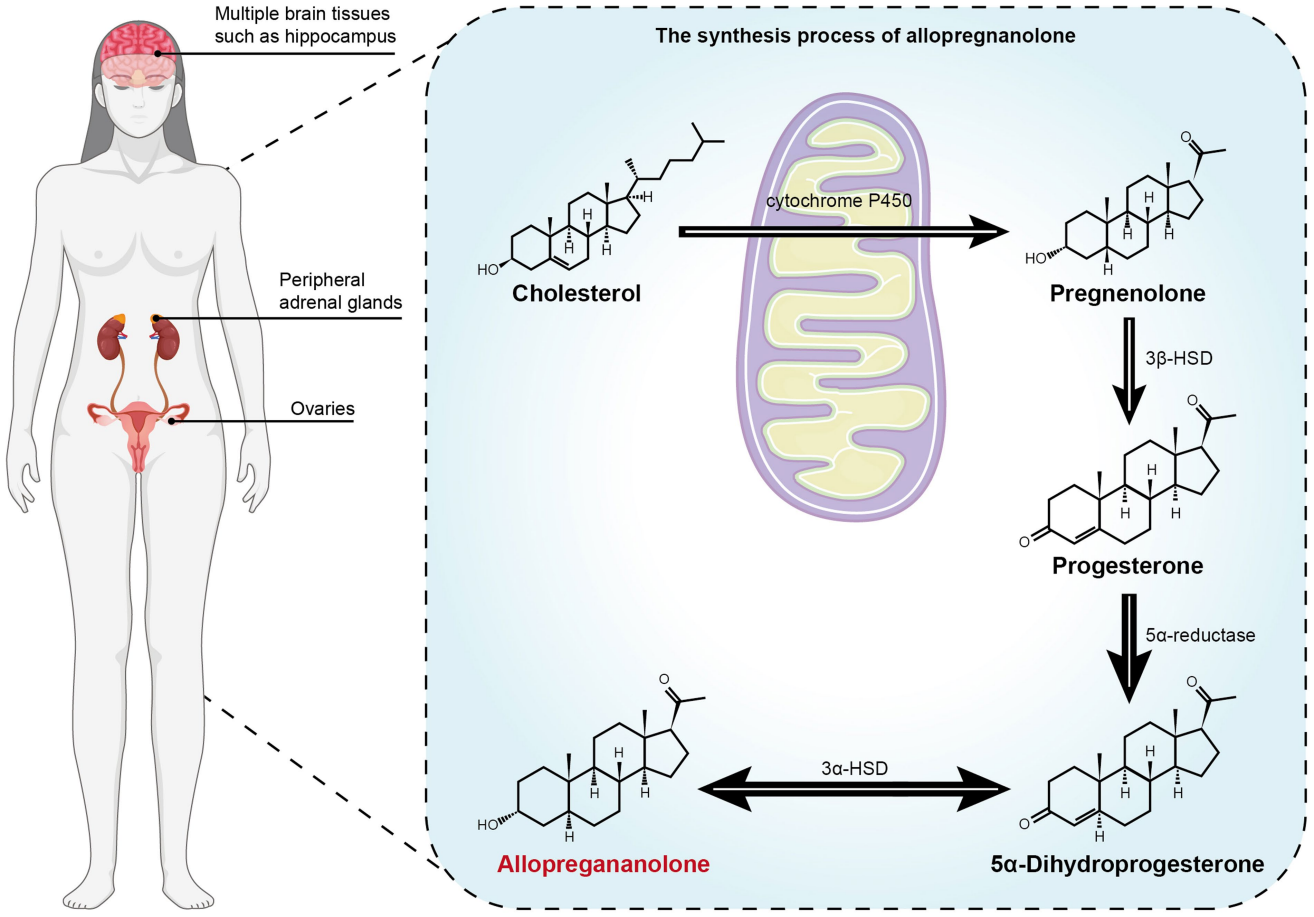
The metabolism process of the precursor cholesterol to allopregnanolone. Cholesterol is transported to the inner mitochondrial membrane through the 18-kDa transport protein TSPO, and then through the cytochrome P450 cholesterol side-chain lyase P450 (P450scc) to produce pregnenolone. Progesterone is produced from pregnenolone by 3β-hydroxysteroid dehydrogenase (3β-HSD) metabolism, and then the metabolized products are gradually reduced by 5α-reductase and 3α-HSD (25). Allo is synthesized in brain (such as hippocampus), adrenal gland and gonad (24, 27).

The neurosteroid tetrahydroprogesterone, also known as allopregnanolone (ALLO), is a metabolite of progesterone, and its existence has been verified through the use of human brain tissue and animal experiments (22). At present, both neurons and glial cells are capable of synthesizing neurosteroids including ALLO (23, 24), and their synthesis pathways in the brain are roughly similar to those in the periphery (22): Cholesterol is transported to the inner mitochondrial membrane through the 18-kDa transport protein TSPO, and then through the cytochrome P450 cholesterol side-chain lyase P450 (P450scc) to produce pregnenolone. Progesterone is produced from pregnenolone by 3β-hydroxysteroid dehydrogenase (3β-HSD) metabolism, and then, the metabolized products are gradually reduced by 5α-reductase and 3α-HSD (25–27). The formation process is shown in Figure 1. The serum level of ALLO in women of normal childbearing age ranges from 0.2 to 0.5 nmol/L in the follicular phase, increases to 4 nmol/L in the middle luteal phase (28), and fluctuates in the range of 0.9–2 nmol/L in the late luteal phase (29–31). However, compared with the ALLO concentration corresponding to the normal luteal phase level, both high and low levels of ALLO can cause more severe emotional changes, which illustrates the bimodal effect or inverted U model of change of ALLO on emotion (32).

It has been observed in animal experiments that ALLO has a two-way emotional regulation effect (33). Low-dose ALLO increases the PMDD-like behavior of female mice, while high-dose ALLO reduces aggressive behaviors (34). However, in healthy women, negative emotional reactions triggered by changes in ALLO levels have not been observed (35). One possible reason why ALLO changes cause negative emotions is the plasticity of the GABA_A_ receptor, because the composition and pharmacological properties of GABA_A_ receptor subunits have been shown to change with different reproductive states (36). The GABA_A_ receptor is an ion channel receptor related to ALLO, and ALLO shows high affinity for most GABA_A_ receptor subtypes (37), which can enhance the GABA evoked chloride current through increasing the frequency and/or duration of openings of the GABA-gated chloride channel (38–40).

## 3. Fluctuations in ALLO levels are key triggers for PMDD symptoms

PMDD can be traced back to female puberty, and it is closely related to the periodic changes of female ovarian function. The negative emotional changes of PMDD patients started from ovulation in a menstrual cycle, and gradually presented and significantly increased with the production of corpus luteum, especially about the last 5 days of corpus luteum period, the symptoms gradually relieved or disappeared after menstruation. Clinical studies have gradually revealed that ovarian hormones play an important role in the pathogenesis of PMDD, especially estradiol and progesterone metabolite ALLO, and a series of hormone therapies have been derived from them (41).

Although ALLO plays an important role in PMS/PMDD, its relationship is not very clear. Compared with the normal situation, does the level of ALLO in PMS/PMDD patients increase or decrease? There is no consensus on how the level of ALLO changes in patients with PMDD after cure. Some studies have shown that the remission of PMS/PMDD symptoms is related to the reduction of ALLO level. After treating PMS with sertraline and desipramine, Freeman et al. found that PMS symptoms were related to a reduction in ALLO levels (42). A similar conclusion appeared after the use of the gonadotropin-releasing hormone agonist buserelin to induce ovarian suppression, and it was found that low-dose buserelin can ameliorate negative mood symptoms (43). However, under the environment of excluding the influence of estrogen and simulating progesterone during the luteal phase, the average serum level of ALLO in the PMDD group was not significantly different from that in the control group (44). Nevertheless, TV Nguyen et al. found that the increase of the content of neurosteroids in luteal phase, rather than the basic level, mediates the onset of female symptoms of PMDD, because blocking 5 α Reductase activity can reduce the onset of PMDD symptoms (44). A study using the 5α-reductase inhibitor dutasteride to treat PMDD found that patients who received high doses (2.5 mg/day) of dutasteride experienced relief of the core symptoms of the luteal phase after drug treatment, and the level of ALLO in the luteal phase was lower than that in the follicular phase. In contrast, there were lower levels of ALLO in the follicular phase than in the luteal phase for patients who received low-dose (0.5 mg/day) dutasteride and placebo (45).

Although this study suggests that hindering the conversion of progesterone to ALLO can alleviate the core symptoms of PMDD, in most studies, PMDD symptoms were alleviated when ALLO levels were increased (35, 46). Some studies found that the level of ALLO in women with PMS in the luteal phase was lower than that in healthy women whose ALLO was significantly higher in the luteal phase as compared to the follicular phase (47, 48). However, another study showed that in the follicular phase, there was no significant difference in the levels of ALLO between the PMS group and control group (49). These results showed that the decrease in the ALLO level in the luteal phase may be one of the biological factors that contribute to anger and depression in PMS patients. Some studies divided PMS patients with different ALLO levels into high, medium, and low groups, with 2.55 ng/ml as the baseline (50). After treatment with sertraline, it was found that the change in ALLO levels was associated with specific emotional symptoms, such as depression, loss of control, and helplessness. Sertraline increased the ALLO levels in the low baseline group, and it ameliorated the above-described emotional symptoms. In the middle baseline group, the negative mood symptoms were also ameliorated, but the ALLO level did not significantly change. In the high baseline group, the level of ALLO decreased, with only slight amelioration of symptoms (50). The relevance between ALLO and PMS/PMDD was confirmed by the reversal of ALLO concentration after intervention with anti-depression drugs. The level of ALLO significantly increased in brain tissue after fluoxetine administration (51). The application of low-dose fluoxetine can prevent ALLO from oxidizing to 5α-dihydroprogesterone (5α-DHP) without affecting the reuptake of 5-HT (52). Short-term low-dose fluoxetine intervention in the late luteal phase can increase the concentration of ALLO in the brain and prevent the onset of PMDD (10).

Although the above evidences have different conclusions, it is confirmed that the fluctuation of ovarian steroid hormone level can indeed affect the pathogenesis of PMDD. The luteal phase is both the period of PMDD onset and the period of rapid decrease of progesterone, so the fluctuation of the level of neurosteroids may be the key trigger factor affecting the onset of PMDD (53). At the same time, Yan Li proposed the animal model of PMDD based on withdrawal of progesterone or ALLO (54). In rodent experiments, the rapid withdrawal of ALLO will produce symptoms characterized by PMDD, such as increased anxiety behavior (increased acute start response, decreased the opening arm entry time in the elevated plus-maze test) (55) and depressive behavior (increased suspension immobility time in forced swimming test, social withdrawal in social preference test, and lack of pleasure in sugar water preference test) (54).

## 4. ALLO-mediated GABA_A_ receptor sensitivity is involved in key mechanisms of PMDD

### 4.1. Physiological properties of the GABA_A_ receptor

The GABA_A_ receptor is a transmembrane protein complex. After binding with GABA or the appropriate agonists, it can play a regulatory role by regulating the flow of chloride ions, so as to hyperpolarize neurons. GABA_A_ receptors are composed of 19 subunits (α1–α6, β1–β3, γ1–γ3, δ1, ε1, θ, π1, and ρ1–ρ3) from eight subunit families (56, 57). The assembling of different subunits results in different receptor characteristics in different brain regions. The subunits closely related to PMDD are mainly α, δ, and γ subunits. Receptors containing the α4, α6, and δ subunits mediate persistent tonic suppression in low extrasynaptic concentrations of GABA. In high post-synaptic GABA concentrations, the α1, α2, α3, and γ subunit receptors mediate rapid and short-term phase inhibition (58–60). Compared with postsynaptic receptors that require high concentrations of GABA to activate, there is a lower activation threshold for extrasynaptic receptors. ALLO can enhance phasic and tonic inhibition by combining synapses and extrasynaptic GABA_A_ receptors (37). Under physiological conditions, GABA_A_ receptor activation hyperpolarized the neurons and inhibited the excitability through to influx of chloride ions. The tonic inhibitory current produces a greater amount of charge transfer, and thus, regulating the expression of receptors containing the α4, α6, and δ subunits is an effective method to reduce the excitability of neurons (61).

### 4.2. The key evidence of interaction between ALLO and GABA_A_ receptor- saccadic eye velocity

Saccadic eye velocity (SEV) is rapid, steplike, conjugate changes of gaze, the purpose of which is calculate the speed of centralizing objects of interest on the fovea (62). SEV is controlled by frontal lobe, substantia nigra, superior colliculus, pontine reticular formation, and cerebellum, and there are a lot of GABA_A_ receptors in these regions. It has been shown to be a reliable neurophysiological tool for the assessment of GABA_A_ receptor sensitivity (63). Therefore, the results of SEV experiment can be used as evidence for the interaction between ALLO and GABA_A_ receptor. Allopregnanolone has been shown to dose-dependently decrease SEV and increase subjective sedation in humans (64). Therefore, the sensitivity of GABA_A_ receptor related to the fluctuation of ALLO may be the cause of PMDD.

### 4.3. Altered GABA_A_ receptor sensitivity in PMDD pathogenesis

#### 4.3.1. ALLO affects the expression of GABA_A_ receptor subunits

The expression of GABA_A_ receptor subunits, such as α4, may be a key point under PMDD pathophysiology (65–67). The expression of the extrasynaptic GABA_A_ receptor is significantly affected by fluctuations in the concentration of extracellular ALLO (10). The expression of the δ subunit decreased after intervention with the 5α-reductase inhibitor finasteride (68). Before fluoxetine administration, GABA decreased, and expression of the α4 subunit increased in the brain area of rats developed as a PMDD model. After fluoxetine administration, the ALLO concentration and α4 subunit expression level reversed (69, 70). After progesterone withdrawal in a PMDD rat model, it was observed that the expression of the α4 and δ subunits in the brain increased, which led to changes in neuronal excitability and induced PMDD-like behavior in rats (10). It was also observed that the mRNA expression of the GABA_A_ receptor α4 and δ subunits decreased in the brain of depressed patients (71). Recently found in the studies of rats have found that the periaqueductal gray (PAG) substance, which is involved in the regulation of fear responses, is involved in the pathogenesis of PMDD (72). GABAergic neurons are widely distributed in the dorsolateral area of the PAG. The progesterone level in female rats sharply decreased in the late estrus period, and the expression of GABA_A_ receptor α4, β1, and δ subunits in the PAG area was upregulated, which enhances the excitability of neural circuits in the PAG area and induces anxiety and panic (73).

The α4, β, and δ subunits of the GABA_A_ receptor are mainly expressed in the dentate gyrus and thalamus (28). The effect of upregulation of subunit expression on granule cells of the dentate gyrus was significantly higher than that on pyramidal cells of the CA1 region (68). Electrophysiology also confirmed that the GABA current mediated by ALLO enhancement in granule cells of the dentate gyrus was higher than that in pyramidal cells of the CA1 region (68). It has been reported that the increased expression of the δ subunit in the striatum may be a protective mechanism that compensates for the increased excitability of neurons (74).

ALLO has a strong mediating effect on the expression of the δ and α4 subunits of the GABA_A_ receptor (17, 37). It is currently known that there are two types of binding sites of ALLO on the GABA_A_ receptor: (1) one that enhances the effects of steroids and is in the α subunit in the cavity of the basal transmembrane structure and (2) one that directly activates the receptor-gated channel and is located between the α and β subunit interfaces (75–78). ALLO works by enhancing sites at low levels and can directly activate receptors at high levels. Although there is no research suggesting that the ALLO binding site is related to the δ subunit, the ability of ALLO to potentiate GABAA receptors is greater when these receptors contain the delta subunit (79, 80).

#### 4.3.2. ALLO affects the function of GABA_A_ receptor subunits

The negative emotional symptoms of women with PMDD are caused by the contradictory effects of the change in the GABA_A_ receptor mediated by ALLO (81). Martinez et al. (45) proposed that it is the changes in the level of neurosteroids that cause the adjustment in the GABA_A_ receptor. The regulation of ALLO on the receptors is inseparable from the plasticity of the receptors themselves. The plasticity of the GABA_A_ receptor refers to the selective changes in the composition of subunits in different regions due to the changes in the reproductive cycle (64, 68).

Animal experiments have confirmed that the expression of the GABA_A_ receptor subunits periodically changes under physiological conditions. The expression of the δ subunit protein and mRNA in the high progesterone period is higher than that in the low progesterone period, especially in the hippocampal dentate gyrus (68). In the PMDD animal model, it was observed that the expression of the α4 subunit in the hippocampus was upregulated, the affinity for benzodiazepines decreased, and anxiety-like behaviors appeared at the same time (82). It is known that the δ subunits preferentially combine with α4 subunits, which contain benzodiazepine binding sites between the α and γ subunit interface. Therefore, the increase in the expression of the α4 and δ subunits leads to a decrease in the receptor affinity for benzodiazepines due to the γ subunit being replaced by the δ subunit and suppressing the release of GABA, resulting in PMDD-like behavior in rats (83).

The plasticity of the GABA_A_ receptor is impaired and its receptor subunits cannot adapt to the fluctuation of ALLO during the menstrual cycle, resulting in the pathogenesis of PMDD (17). Neurophysiological experiments showed that expression of the GABA_A_ receptor α4, β2, and δ subunits increased 30 min after treatment with ALLO and GABA, and reached a peak after 48 h, while expression of the GABA_A_ receptor α4, β2, and δ subunits on the cell membrane surface did not increase 48 h after treatment with GABA alone (61). GABA acts as a partial agonist on the GABA_A_ receptor α4, β2, and δ subunits, and ALLO can increase the potency of GABA acting on the receptor, which may explain the high affinity of ALLO for the α4 and δ subunits (61, 76). Therefore, the sensitivity of the GABA_A_ receptor to ALLO refers to the ability to respond to fluctuations in ALLO levels. The compositional conditions of this response ability include GABA_A_ receptor affinity for ALLO, and the inherent plasticity of the receptor.

Summarizing the above studies, the following assumptions are put forward: due to the high affinity of ALLO for the extrasynaptic GABA_A_ receptor, when the level of ALLO decreases, the expression of the α4, β, and δ subunits increases. When the level of ALLO increases, the α4, β, and δ subunits do not return to normal and show impaired plasticity. This mediates the effect of ALLO on the receptors and causes the inhibitory neurons to be inhibited. Neurons exhibit disinhibition and a net effect of excitability, which then affects emotional changes. The changes in ALLO levels affect the expression of the α4 and δ subunits, which leads to increased sensitivity of the extrasynaptic GABA_A_ receptor to ALLO. One of the results is that ALLO becomes a trigger point that easily affects women’s moods and can lead to disorders. This result is not only related to genetics, but is also subject to the effects of stress (84).

## 5. ALLO-mediated GABA_A_ receptor: The most viable potential drug target for alleviating PMDD

At present, there are mainly two types of clinical drugs targeting GABA_A_ receptors: GABA_A_ receptor-regulated steroid antagonists (sepranolone) and GABA_A_ receptor-selective positive allosteric modulators [brexanolone, ganaxolone, and SAGE-217 (zuranolone)].

Sepranolone (or isoallopregnanolone) is a metabolite of ALLO that is a GABA_A_ receptor-modulating steroid antagonist that can selectively inhibit the enhancement of GABA_A_ receptor-induced current mediated by neurosteroids on the GABA_A_ receptor. Currently, phase II clinical trials for PMDD are underway. In preclinical studies, it has been shown that sepranolone inhibits the effect of ALLO on GABA_A_ receptor chloride uptake mediated by GABA *in vitro* (85). Satisfactory clinical effects have been obtained with the drug and high safety. Emotional symptoms were satisfactorily relieved, but there was no effect on physical symptoms (17). There were no obvious side effects in clinical trials. The only thing to be noted is that subcutaneous administration produced injection site reactions during the luteal phase (86).

GABA_A_ receptor-selective positive allosteric modulator antidepressants can cause conformational changes in receptors and regulate the affinity of receptors for GABA. Examples of such antidepressants are brexanolone, ganaxolone, and SAGE-217 (zuranolone). Brexanolone (allopregnanolone) is approved by the FDA for postpartum depression (PPD). The pathogenesis of PPD is similar to that of PMDD. The rapid decline of ALLO levels is considered to be the cause of its pathogenesis, and ALLO is the key factor (87). Clinical trials have shown that brexanolone can significantly reduce the depression score of patients with moderate to severe PPD. The most common side effects are headache, dizziness, and sleepiness (16, 88). This drug is used to treat PPD through intravenous infusion for 60 h under continuous medical supervision because of the risks of severe sedation, hypnosis, loss of consciousness, and deep respiratory arrest (89). Ganaxolone and SAGE-217 (zuranolone) are synthetic analogs of ALLO. Ganaxolone is a synthetic 3β-methyl ALLO derivative with sedative effects that can be used as an adjuvant drug for PMDD (90). Preclinical studies have shown that SAGE-217 can exhibit 30–60% oral bioavailability in rodents and demonstrate clear pharmacodynamic effects, consistent with GABA_A_ receptor activity, following oral administration (91). In a phase I clinical trial, oral administration of SAGE-217 was well tolerated without severe adverse reactions (92). In a double-blind phase II clinical trial, MDD patients treated with SAGE-217 for 14 days exhibited significantly reduced depressive symptoms (71). At present, SAGE-217 has entered a number of phase II clinical trials and is expected to become a new drug for the treatment of major depression and PMDD. In conclusion, the ALLO-GABA_A_ receptor remains as the most promising target for the treatment of PMDD.

## 6. Conclusion and prospects

**Figure 2:**
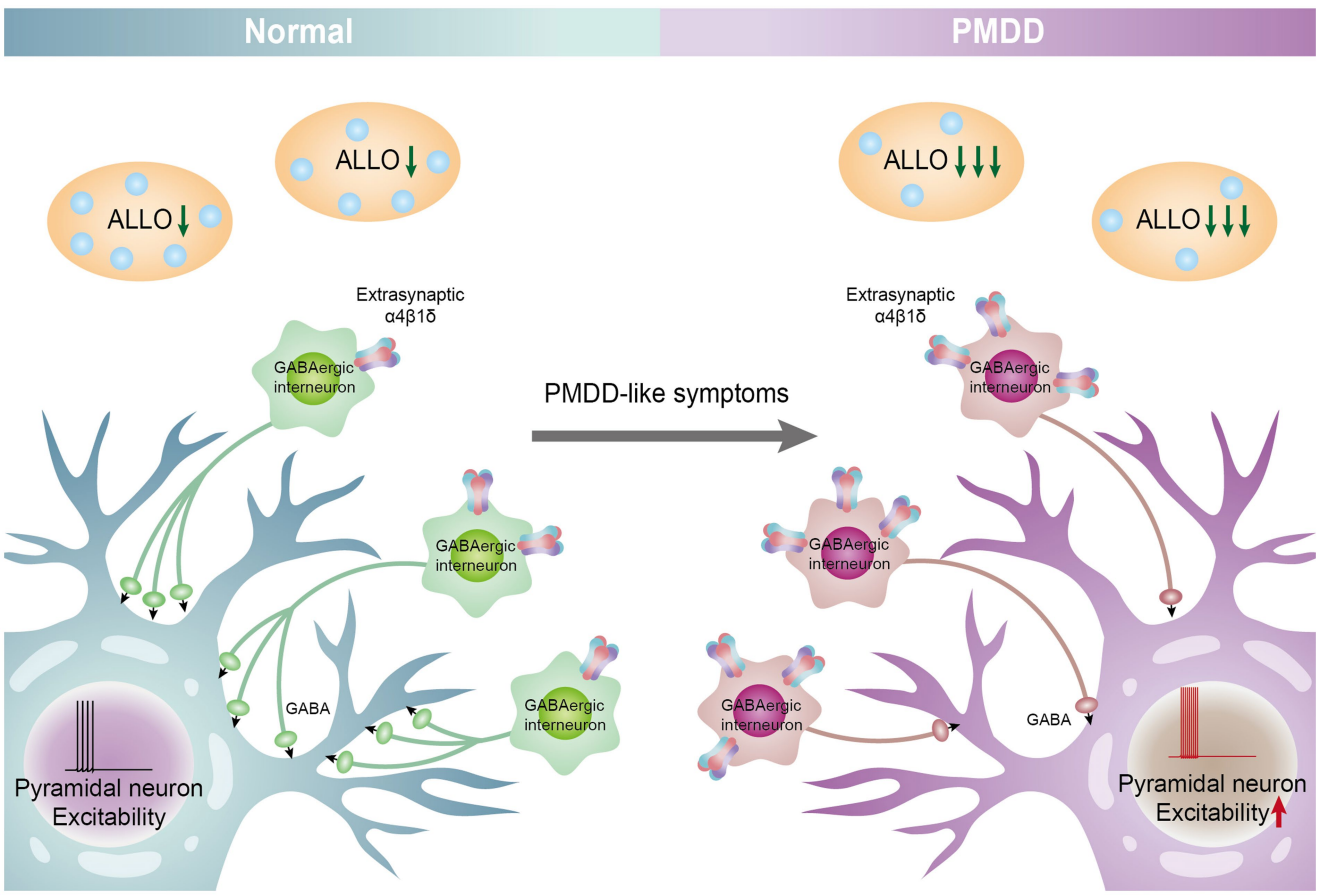
ALLO-mediated GABA_A_ receptor subunit sensitivity participates in the pathogenesis of PMDD. When the decrease in ALLO is too rapid, there is an increase in the expression of GABA_A_ receptor α4 β subunits (10) and decreases in the sensitivity (decreased affinity, reduced plasticity), and leading to a decrease in chloride influx, which, in turn, inhibits the release of GABA from GABAergic interneurons, reduces the inhibition of pyramidal neurons, and then increases the excitability of pyramidal neurons, leading to the development of PMDD. The ALLO-mediated GABA_A_ receptor remains the main pathogenic factor of PMDD.

ALLO is strongly associated with some specific female mental disorders, such as PMS/PMDD (93), catamenial epilepsy (94), menstrually related and postmenopausal migraine (95), and PPD (16, 96). At present, there are few clinical and animal experiments, and the specific mechanism of action is still unclear. After summarizing the above contents, we can draw a conjecture: when the decrease in ALLO is too rapid, there is an increase in the expression of GABAA receptor subunits and decrease in the sensitivity (decreased affinity, reduced plasticity), leading to a decrease in chloride influx, which, in turn, inhibits the release of GABA from GABAergic interneurons, reduces the inhibition of pyramidal neurons, and then increases the excitability of pyramidal neurons, leading to the development of PMDD. The ALLO-mediated GABAA receptor remains the main pathogenic factor of PMDD (Figure 2). ALLO is a positive allosteric modulator of GABA_A_ receptor. When ALLO binds to GABA_A_ receptor, it will enhance the GABA evoked chloride current through increasing the frequency and/or duration of openings of the GABA-gated chloride channel (40). Under normal physiological conditions, GABA_A_ receptor changes normally with the fluctuation of ALLO in the menstrual cycle. When ALLO decreases too fast, the binding rate of ALLO and GABA_A_ receptor decreases, or the plasticity of GABA_A_ receptor is impaired, leading to the decrease of chloride influx. GABAergic interneurons release less GABA, reducing the inhibition of pyramidal neurons (disinhibition of inhibitory neurons), enhancing the excitability of pyramidal neurons, and leading to the occurrence of PMDD. ALLO-mediated GABA_A_ receptor sensitivity is still the main pathogenic factor of PMDD. GABA_A_ receptor (conformation and affinity to GABA), ALLO (elevated level), and the change of GABA_A_ receptor mediated by ALLO receptor may be the therapeutic target of PMDD. Based on the study of changes in ALLO levels related to PMDD, the following conclusions are drawn: (1) The changes in the levels of ALLO participate in the onset of PMDD as a trigger factor for emotional symptoms; (2) The absolute level of ALLO is not the conclusive cause of the onset of PMDD, but rather, the focus should be on the GABA_A_ receptor; (3) The level of ALLO cause changes in the expression and function of GABA_A_ receptor subunits in the brain, which results in increasing sensitivity to ALLO in women with PMDD (97). The sensitivity of the GABA_A_ receptor for ALLO fluctuations refers to the affinity of the GABA_A_ receptor to ALLO and the plasticity of the receptor. The rapid decline of ALLO leads to the decrease of the affinity between ALLO and GABA_A_ receptor and the impairment of the plasticity of GABA_A_ receptor. Therefore, PMDD leads to the decrease of sensitivity of GABA_A_ receptor to ALLO fluctuation and the increase of pressure sensitivity; and (4) Women with PMDD change their response to ALLO under stress.

A goal of future research is to deeply understand the mechanism related to the GABA receptor and ALLO and deeply explore this mechanism so as to develop more effective therapeutic drugs with fewer side effects. However, because the interventions for the treatment of PMDD are mainly chemical drugs, their side effects are extensive. Complementary and alternative medicine represented by traditional Chinese medicine has unique advantages in the treatment of emotional diseases. TCM compounds and active ingredients can act on brain areas that control emotions *via* multiple targets and channels, and acupuncture can activate specific neural circuits and neuroendocrine pathways, thereby ameliorating PMDD symptoms. In the future, research in this field can be directed toward revealing the neural circuit and neuroendocrine mechanism of Chinese herbal compound in the pathogenesis of PMDD and clarified the interventional pathways and targets of complementary and alternative therapies at this level. Then, we will focus on the main components that may play a role in the drug effect. Through the isotope labeling method/virus tracing method, we will deeply explore whether the effective components play a role by influencing the GABAergic nervous system, and how the specific mechanism is. In short, GABA_A_ receptor is a very potential target. Comprehensive exploration in this field will further reveal the possible neurobiological mechanism, thus promoting the development of translational medicine and drugs.

## Author contributions

QG, WS, and Y-RW: writing original draft. Z-FL: data collection. FZ and X-WG: writing—reviewing and editing. K-YX and DC: conceptualization and methodology. KL and YX: visualization. WL and SW: project administration, supervision, and funding acquisition. All authors contributed to the article and approved the submitted version.

## Funding

This study was supported by the National Natural Science Foundation of China (Nos. 82274383, 82004078, and 81974553), the Natural Science Foundation of Shandong Province (Nos. ZR2020ZD17 and ZR2021LZY018), the Special Funding for Taishan Scholars Project (No. tsqn202211137), the High-Caliber TCM Talents Training Program of Shandong Province and Mainland China, the Chinese Medicine and Brain Science Youth Scientific Research Innovation Team, and the Shandong University of Traditional Chinese Medicine (No. 22202101).

## Conflict of interest

The authors declare that the research was conducted in the absence of any commercial or financial relationships that could be construed as a potential conflict of interest.

## Publisher’s note

All claims expressed in this article are solely those of the authors and do not necessarily represent those of their affiliated organizations, or those of the publisher, the editors and the reviewers. Any product that may be evaluated in this article, or claim that may be made by its manufacturer, is not guaranteed or endorsed by the publisher.

## References

[1] Noviyanti NI Gusriani R Mappaware NA Ahmad M. The effect of estrogen hormone on premenstrual syndrome (PMS) occurrences in teenage girls at Pesantren Darul Arqam Makassar. Gac Sanit. (2021) 35:S571–5. doi: 10.1016/j.gaceta.2021.10.103 34929904

[2] Yen JY Wang PW Su CH Liu TL Long CY Ko CH. Estrogen levels, emotion regulation, and emotional symptoms of women with premenstrual dysphoric disorder: the moderating effect of estrogen receptor 1alpha polymorphism. Prog Neuro-Psychopharmacol Biol Psychiatry. (2018) 82:216–23. doi: 10.1016/j.pnpbp.2017.11.013, PMID: 29146473

[3] Beddig T Reinhard I Kuehner C. Stress, mood, and cortisol during daily life in women with premenstrual dysphoric disorder (PMDD). Psychoneuroendocrinology. (2019) 109:104372. doi: 10.1016/j.psyneuen.2019.104372 31357135

[4] Battle DE. Diagnostic and statistical manual of mental disorders (DSM). Codas. (2013) 25:191–2. doi: 10.1590/s2317-17822013000200017, PMID: 24413388

[5] Epperson CN Steiner M Hartlage SA Eriksson E Schmidt PJ Jones I. Premenstrual dysphoric disorder: evidence for a new category for DSM-5. Am J Psychiatry. (2012) 169:465–75. doi: 10.1176/appi.ajp.2012.11081302, PMID: 22764360 PMC3462360

[6] Halbreich U Borenstein J Pearlstein T Kahn LS. The prevalence, impairment, impact, and burden of premenstrual dysphoric disorder (PMS/PMDD). Psychoneuroendocrinology. (2003) 28:1–23. doi: 10.1016/s0306-4530(03)00098-2 12892987

[7] Soyak HM Acmaz G Uguz F. Investigation of premenstrular dysphoric disorder comorbidity and related factors in patients with anxiety disorder. Ann Med Res. (2021) 28:1870–6. doi: 10.5455/annalsmedres.2020.11.1117, PMID: 34463974

[8] Hong JP Park S Wang HR Chang SM Sohn JH Jeon HJ. Prevalence, correlates, comorbidities, and suicidal tendencies of premenstrual dysphoric disorder in a nationwide sample of Korean women. Soc Psychiatry Psychiatr Epidemiol. (2012) 47:1937–45. doi: 10.1007/s00127-012-0509-6, PMID: 22538387

[9] Marjoribanks J Brown J O'Brien PM Wyatt K. Selective serotonin reuptake inhibitors for premenstrual syndrome. Cochrane Database Syst Rev. (2013) 2013:CD001396. doi: 10.1002/14651858.CD001396.pub3, PMID: 23744611 PMC7073417

[10] Lovick T. SSRIs and the female brain--potential for utilizing steroid-stimulating properties to treat menstrual cycle-linked dysphorias. J Psychopharmacol. (2013) 27:1180–5. doi: 10.1177/0269881113490327, PMID: 23704364

[11] Yonkers KA O'Brien PM Eriksson E. Premenstrual syndrome. Lancet. (2008) 371:1200–10. doi: 10.1016/S0140-6736(08)60527-9, PMID: 18395582 PMC3118460

[12] Halbreich U O'Brien PM Eriksson E Bäckström T Yonkers KA Freeman EW. Are there differential symptom profiles that improve in response to different pharmacological treatments of premenstrual syndrome/premenstrual dysphoric disorder? CNS Drugs. (2006) 20:523–47. doi: 10.2165/00023210-200620070-00001 16800714

[13] Lochmann D Richardson T. Selective serotonin reuptake inhibitors. Handb Exp Pharmacol. (2019) 250:135–44. doi: 10.1007/164_2018_172, PMID: 30838457

[14] Hantsoo L Epperson CN. Premenstrual dysphoric disorder: epidemiology and treatment. Curr Psychiatry Rep. (2015) 17:87. doi: 10.1007/s11920-015-0628-3 26377947 PMC4890701

[15] Sundström-Poromaa I Comasco E Sumner R Luders E. Progesterone – friend or foe? Front Neuroendocrinol. (2020) 59:100856. doi: 10.1016/j.yfrne.2020.100856, PMID: 32730861

[16] Meltzer-Brody S Kanes SJ. Allopregnanolone in postpartum depression: role in pathophysiology and treatment. Neurobiol Stress. (2020) 12:100212. doi: 10.1016/j.ynstr.2020.100212, PMID: 32435663 PMC7231991

[17] Hantsoo L Epperson CN. Allopregnanolone in premenstrual dysphoric disorder (PMDD): evidence for dysregulated sensitivity to GABA-A receptor modulating neuroactive steroids across the menstrual cycle. Neurobiol Stress. (2020) 12:100213. doi: 10.1016/j.ynstr.2020.100213, PMID: 32435664 PMC7231988

[18] Wenzel ES Pinna G Eisenlohr-Moul T Bernabe BP Tallon RR Nagelli U. Neuroactive steroids and depression in early pregnancy. Psychoneuroendocrinology. (2021) 134:105424. doi: 10.1016/j.psyneuen.2021.105424, PMID: 34607173 PMC8943472

[19] Rabinowitz A Cohen SJ Finn DA Stackman RW Jr. The neurosteroid allopregnanolone impairs object memory and contextual fear memory in male C57BL/6J mice. Horm Behav. (2014) 66:238–46. doi: 10.1016/j.yhbeh.2014.05.005, PMID: 24874172

[20] Majewska MD Schwartz RD. Pregnenolone-sulfate: an endogenous antagonist of the gamma-aminobutyric acid receptor complex in brain? Brain Res. (1987) 404:355–60. 3032339 10.1016/0006-8993(87)91394-1

[21] Longone P Rupprecht R Manieri GA Bernardi G Romeo E Pasini A. The complex roles of neurosteroids in depression and anxiety disorders. Neurochem Int. (2008) 52:596–601. doi: 10.1016/j.neuint.2007.10.001 17996986

[22] Baulieu EE Robel P. Neurosteroids: a new brain function? J Steroid Biochem Mol Biol. (1990) 37:395–403. doi: 10.1016/0960-0760(90)90490-C, PMID: 2257243

[23] Agís-Balboa RC Pinna G Zhubi A Maloku E Veldic M Costa E. Characterization of brain neurons that express enzymes mediating neurosteroid biosynthesis. Proc Natl Acad Sci U S A. (2006) 103:14602–7. doi: 10.1073/pnas.0606544103, PMID: 16984997 PMC1600006

[24] Diviccaro S Cioffi L Falvo E Giatti S Melcangi RC. Allopregnanolone: an overview on its synthesis and effects. J Neuroendocrinol. (2022) 34:e12996. doi: 10.1111/jne.12996, PMID: 34189791 PMC9285581

[25] Dichtel LE Lawson EA Schorr M Meenaghan E Paskal ML Eddy KT. Neuroactive steroids and affective symptoms in women across the weight Spectrum. Neuropsychopharmacology. (2018) 43:1436–44. doi: 10.1038/npp.2017.269, PMID: 29090684 PMC5916351

[26] Mellon SH Griffin LD Compagnone NA. Biosynthesis and action of neurosteroids. Brain Res Brain Res Rev. (2001) 37:3–12. doi: 10.1016/s0165-0173(01)00109-6 11744070

[27] Stoffel-Wagner B. Neurosteroid biosynthesis in the human brain and its clinical implications. Ann N Y Acad Sci. (2003) 1007:64–78. doi: 10.1196/annals.1286.007 14993041

[28] Wang M. Neurosteroids and GABA-A receptor function. Front Endocrinol. (2011) 2:44. doi: 10.3389/fendo.2011.00044 PMC3356040 22654809

[29] Wang M Seippel L Purdy RH Bãckström T. Relationship between symptom severity and steroid variation in women with premenstrual syndrome: study on serum pregnenolone, pregnenolone sulfate, 5 alpha-pregnane-3,20-dione and 3 alpha-hydroxy-5 alpha-pregnan-20-one. J Clin Endocrinol Metab. (1996) 81:1076–82. doi: 10.1210/jcem.81.3.8772579, PMID: 8772579

[30] Genazzani AR Petraglia F Bernardi F Casarosa E Salvestroni C Tonetti A. Circulating levels of allopregnanolone in humans: gender, age, and endocrine influences. J Clin Endocrinol Metab. (1998) 83:2099–103. doi: 10.1210/jcem.83.6.4905, PMID: 9626145

[31] Kimball A Dichtel LE Nyer MB Mischoulon D Fisher LB Cusin C. The allopregnanolone to progesterone ratio across the menstrual cycle and in menopause. Psychoneuroendocrinology. (2020) 112:104512. doi: 10.1016/j.psyneuen.2019.104512, PMID: 31780185 PMC6935417

[32] Bixo M Ekberg K Poromaa IS Hirschberg AL Jonasson AF Andreen L. Treatment of premenstrual dysphoric disorder with the GABAA receptor modulating steroid antagonist Sepranolone (UC1010)-a randomized controlled trial. Psychoneuroendocrinology. (2017) 80:46–55. doi: 10.1016/j.psyneuen.2017.02.031, PMID: 28319848

[33] Locci A Pinna G. Neurosteroid biosynthesis down-regulation and changes in GABA(A) receptor subunit composition: a biomarker axis in stress-induced cognitive and emotional impairment. Br J Pharmacol. (2017) 174:3226–41. doi: 10.1111/bph.13843, PMID: 28456011 PMC5595768

[34] Fish EW Faccidomo S DeBold JF Miczek KA. Alcohol, allopregnanolone and aggression in mice. Psychopharmacology. (2001) 153:473–83. doi: 10.1007/s002130000587, PMID: 11243495

[35] McEvoy K Osborne LM. Allopregnanolone and reproductive psychiatry: an overview. Int Rev Psychiatry. (2019) 31:237–44. doi: 10.1080/09540261.2018.1553775, PMID: 30701996 PMC6594874

[36] Rapkin AJ Korotkaya Y Taylor KC. Contraception counseling for women with premenstrual dysphoric disorder (PMDD): current perspectives. Open Access J Contracept. (2019) 10:27–39. doi: 10.2147/OAJC.S183193, PMID: 31572029 PMC6759213

[37] Carver CM Reddy DS. Neurosteroid interactions with synaptic and extrasynaptic GABA(A) receptors: regulation of subunit plasticity, phasic and tonic inhibition, and neuronal network excitability. Psychopharmacology. (2013) 230:151–88. doi: 10.1007/s00213-013-3276-5, PMID: 24071826 PMC3832254

[38] Gonzalez SL Coronel MF Raggio MC Labombarda F. Progesterone receptor-mediated actions and the treatment of central nervous system disorders: an up-date of the known and the challenge of the unknown. Steroids. (2020) 153:108525. doi: 10.1016/j.steroids.2019.108525, PMID: 31634489

[39] Chen ZW Bracamontes JR Budelier MM Germann AL Shin DJ Kathiresan K. Multiple functional neurosteroid binding sites on GABAA receptors. PLoS Biol. (2019) 17:e3000157. doi: 10.1371/journal.pbio.3000157, PMID: 30845142 PMC6424464

[40] Rupprecht R. Neuroactive steroids: mechanisms of action and neuropsychopharmacological properties. Psychoneuroendocrinology. (2003) 28:139–68. doi: 10.1016/S0306-4530(02)00064-1, PMID: 12510009

[41] Wyatt KM Dimmock PW Ismail KM Jones PW O'Brien PM. The effectiveness of GnRHa with and without 'add-back' therapy in treating premenstrual syndrome: a meta analysis. BJOG. (2004) 111:585–93. doi: 10.1111/j.1471-0528.2004.00135.x, PMID: 15198787

[42] Freeman EW Frye CA Rickels K Martin PA Smith SS. Allopregnanolone levels and symptom improvement in severe premenstrual syndrome. J Clin Psychopharmacol. (2002) 22:516–20. doi: 10.1097/00004714-200210000-00013, PMID: 12352277

[43] Nyberg S Backstrom T Zingmark E Purdy RH Poromaa IS. Allopregnanolone decrease with symptom improvement during placebo and gonadotropin-releasing hormone agonist treatment in women with severe premenstrual syndrome. Gynecol Endocrinol. (2007) 23:257–66. doi: 10.1080/09513590701253511 17558683

[44] Nguyen TV Reuter JM Gaikwad NW Rotroff DM Kucera HR Motsinger-Reif A. The steroid metabolome in women with premenstrual dysphoric disorder during GnRH agonist-induced ovarian suppression: effects of estradiol and progesterone addback. Transl Psychiatry. (2017) 7:e1193. doi: 10.1038/tp.2017.146, PMID: 28786978 PMC5611719

[45] Martinez PE Rubinow DR Nieman LK Koziol DE Morrow AL Schiller CE. 5alpha-reductase inhibition prevents the luteal phase increase in plasma Allopregnanolone levels and mitigates symptoms in women with premenstrual dysphoric disorder. Neuropsychopharmacology. (2016) 41:1093–102. doi: 10.1038/npp.2015.246, PMID: 26272051 PMC4748434

[46] Hantsoo L Grillon C Sammel M Johnson R Marks J Epperson CN. Response to sertraline is associated with reduction in anxiety-potentiated startle in premenstrual dysphoric disorder. Psychopharmacology. (2021) 238:2985–97. doi: 10.1007/s00213-021-05916-6, PMID: 34292344 PMC11146287

[47] Monteleone P Luisi S Tonetti A Bernardi F Genazzani AD Luisi M. Allopregnanolone concentrations and premenstrual syndrome. Eur J Endocrinol. (2000) 142:269–73. doi: 10.1530/eje.0.1420269, PMID: 10700721

[48] Lombardi I Luisi S Quirici B Monteleone P Bernardi F Liut M. Adrenal response to adrenocorticotropic hormone stimulation in patients with premenstrual syndrome. Gynecol Endocrinol. (2004) 18:79–87. doi: 10.1080/09513590310001652955 15195499

[49] Gao H Xia T Qiao MQ. Correlation between neurotransmitters and neurosteroids and premenstrual syndrome patients of Gan-yang ascending syndrome and Gan-qi stagnation syndrome. Zhongguo Zhong Xi Yi Jie He Za Zhi. (2012) 32:1503–7. PMID: 23359974

[50] Gracia CR Freeman EW Sammel MD Lin H Sheng L Frye C. Allopregnanolone levels before and after selective serotonin reuptake inhibitor treatment of premenstrual symptoms. J Clin Psychopharmacol. (2009) 29:403–5. doi: 10.1097/JCP.0b013e3181ad8825, PMID: 19593190

[51] Devall AJ Santos JM Fry JP Honour JW Brandao ML Lovick TA. Elevation of brain allopregnanolone rather than 5-HT release by short term, low dose fluoxetine treatment prevents the estrous cycle-linked increase in stress sensitivity in female rats. Eur Neuropsychopharmacol. (2015) 25:113–23. doi: 10.1016/j.euroneuro.2014.11.017, PMID: 25498416

[52] Fry JP Li KY Devall AJ Cockcroft S Honour JW Lovick TA. Fluoxetine elevates allopregnanolone in female rat brain but inhibits a steroid microsomal dehydrogenase rather than activating an aldo-keto reductase. Br J Pharmacol. (2014) 171:5870–80. doi: 10.1111/bph.12891, PMID: 25161074 PMC4290723

[53] Martinez PE Rubinow DR Nieman LK Koziol DE Morrow AL Schiller CE. 5α-reductase inhibition prevents the luteal phase increase in plasma Allopregnanolone levels and mitigates symptoms in women with premenstrual dysphoric disorder. Neuropsychopharmacology. (2016) 41:1093–102. doi: 10.1038/npp.2015.246, PMID: 26272051 PMC4748434

[54] Li Y Pehrson AL Budac DP Sánchez C Gulinello M. A rodent model of premenstrual dysphoria: progesterone withdrawal induces depression-like behavior that is differentially sensitive to classes of antidepressants. Behav Brain Res. (2012) 234:238–47. doi: 10.1016/j.bbr.2012.06.034, PMID: 22789402

[55] Gulinello M Smith SS. Anxiogenic effects of neurosteroid exposure: sex differences and altered GABAA receptor pharmacology in adult rats. J Pharmacol Exp Ther. (2003) 305:541–8. doi: 10.1124/jpet.102.045120, PMID: 12606703

[56] Chua HC Chebib M. GABAA receptors and the diversity in their structure and pharmacology. Adv Pharmacol. (2017) 79:1–34. doi: 10.1016/bs.apha.2017.03.003 28528665

[57] Simon J Wakimoto H Fujita N Lalande M Barnard EA. Analysis of the set of GABA(A) receptor genes in the human genome. J Biol Chem. (2004) 279:41422–35. doi: 10.1074/jbc.M401354200, PMID: 15258161

[58] Brickley SG Mody I. Extrasynaptic GABA(A) receptors: their function in the CNS and implications for disease. Neuron. (2012) 73:23–34. doi: 10.1016/j.neuron.2011.12.012, PMID: 22243744 PMC3399243

[59] Mortensen M Patel B Smart TG. GABA potency at GABA(A) receptors found in synaptic and extrasynaptic zones. Front Cell Neurosci. (2011) 6:1. doi: 10.3389/fncel.2012.00001, PMID: 22319471 PMC3262152

[60] Herd MB Belelli D Lambert JJ. Neurosteroid modulation of synaptic and extrasynaptic GABA(A) receptors. Pharmacol Ther. (2007) 116:20–34. doi: 10.1016/j.pharmthera.2007.03.007, PMID: 17531325

[61] Kuver A Shen H Smith SS. Regulation of the surface expression of alpha4beta2delta GABAA receptors by high efficacy states. Brain Res. (2012) 1463:1–20. doi: 10.1016/j.brainres.2012.04.047, PMID: 22609410 PMC3371167

[62] Ball DM Glue P Wilson S Nutt DJ. Pharmacology of saccadic eye movements in man. 1. Effects of the benzodiazepine receptor ligands midazolam and flumazenil. Psychopharmacology. (1991) 105:361–7. doi: 10.1007/BF02244431, PMID: 1665920

[63] Zwanzger P Schule C Eser D Baghai TC Padberg F Ella R. Saccadic eye velocity after selective GABAergic treatment with tiagabine in healthy volunteers. Neuropsychobiology. (2005) 52:147–50. doi: 10.1159/000087845, PMID: 16127281

[64] Timby E Backstrom T Nyberg S Stenlund H Wihlback AN Bixo M. Women with premenstrual dysphoric disorder have altered sensitivity to allopregnanolone over the menstrual cycle compared to controls-a pilot study. Psychopharmacology. (2016) 233:2109–17. doi: 10.1007/s00213-016-4258-1, PMID: 26960697

[65] Smith SS Gong QH Li X Moran MH Bitran D Frye CA. Withdrawal from 3alpha-OH-5alpha-pregnan-20-one using a pseudopregnancy model alters the kinetics of hippocampal GABAA-gated current and increases the GABAA receptor alpha4 subunit in association with increased anxiety. J Neurosci. (1998) 18:5275–84. doi: 10.1523/JNEUROSCI.18-14-05275.1998, PMID: 9651210 PMC6793484

[66] Smith SS Ruderman Y Frye C Homanics G Yuan M. Steroid withdrawal in the mouse results in anxiogenic effects of 3alpha,5beta-THP: a possible model of premenstrual dysphoric disorder. Psychopharmacology. (2006) 186:323–33. doi: 10.1007/s00213-005-0168-3, PMID: 16193334 PMC2887339

[67] Gulinello M Orman R Smith SS. Sex differences in anxiety, sensorimotor gating and expression of the alpha4 subunit of the GABAA receptor in the amygdala after progesterone withdrawal. Eur J Neurosci. (2003) 17:641–8. doi: 10.1046/j.1460-9568.2003.02479.x, PMID: 12581182 PMC2887345

[68] Wu X Gangisetty O Carver CM Reddy DS. Estrous cycle regulation of extrasynaptic delta-containing GABA(A) receptor-mediated tonic inhibition and limbic epileptogenesis. J Pharmacol Exp Ther. (2013) 346:146–60. doi: 10.1124/jpet.113.203653, PMID: 23667248 PMC3684839

[69] Rapkin AJ Akopians AL. Pathophysiology of premenstrual syndrome and premenstrual dysphoric disorder. J Menopause Int. (2012) 18, 52–59. doi: 10.1258/mi.2012.012014 22611222

[70] Pinna G Costa E Guidotti A. Fluoxetine and norfluoxetine stereospecifically and selectively increase brain neurosteroid content at doses that are inactive on 5-HT reuptake. J Psychopharmacol. (2006) 186, 362–72. doi: 10.1007/s00213-005-0213-2, PMID: 16432684

[71] Gunduz-Bruce H Silber C Kaul I Rothschild AJ Riesenberg R Sankoh AJ. Trial of SAGE-217 in patients with major depressive disorder. N Engl J Med. (2019) 381:903–11. doi: 10.1056/NEJMoa1815981, PMID: 31483961

[72] Lovick TA Griffiths JL Dunn SM Martin IL. Changes in GABA(A) receptor subunit expression in the midbrain during the oestrous cycle in Wistar rats. Neuroscience. (2005) 131:397–405. doi: 10.1016/j.neuroscience.2004.11.010, PMID: 15708482

[73] Lovick TA. Plasticity of GABAA receptor subunit expression during the oestrous cycle of the rat: implications for premenstrual syndrome in women. Exp Physiol. (2006) 91:655–60. doi: 10.1113/expphysiol.2005.032342, PMID: 16740643

[74] Santhakumar V Jones RT Mody I. Developmental regulation and neuroprotective effects of striatal tonic GABAA currents. Neuroscience. (2010) 167:644–55. doi: 10.1016/j.neuroscience.2010.02.048, PMID: 20206233 PMC2907073

[75] Locci A Pinna G. Neurosteroid biosynthesis down-regulation and changes in GABAA receptor subunit composition: a biomarker axis in stress-induced cognitive and emotional impairment. Br J Pharmacol. (2017) 174:3226–41. doi: 10.1111/bph.13843, PMID: 28456011 PMC5595768

[76] Akk G Covey DF Evers AS Steinbach JH Zorumski CF Mennerick S. Mechanisms of neurosteroid interactions with GABA(A) receptors. Pharmacol Ther. (2007) 116:35–57. doi: 10.1016/j.pharmthera.2007.03.004, PMID: 17524487 PMC2047817

[77] Hosie AM Wilkins ME da Silva HM Smart TG. Endogenous neurosteroids regulate GABAA receptors through two discrete transmembrane sites. Nature. (2006) 444:486–9. doi: 10.1038/nature05324, PMID: 17108970

[78] Hosie AM Clarke L da Silva H Smart TG. Conserved site for neurosteroid modulation of GABA a receptors. Neuropharmacology. (2009) 56:149–54. doi: 10.1016/j.neuropharm.2008.07.050, PMID: 18762201

[79] Seljeset S Laverty D Smart TG. Inhibitory neurosteroids and the GABAA receptor. Adv Pharmacol. (2015) 72:165–87. doi: 10.1016/bs.apha.2014.10.006 25600370

[80] Belelli D Phillips GD Atack JR Lambert JJ. Relating neurosteroid modulation of inhibitory neurotransmission to behaviour. J Neuroendocrinol. (2022) 34:e13045. doi: 10.1111/jne.13045, PMID: 34644812

[81] Backstrom T Bixo M Johansson M Nyberg S Ossewaarde L Ragagnin G. Allopregnanolone and mood disorders. Prog Neurobiol. (2014) 113:88–94. doi: 10.1016/j.pneurobio.2013.07.005, PMID: 23978486

[82] Gulinello M Gong QH Li X Smith SS. Short-term exposure to a neuroactive steroid increases alpha4 GABA(A) receptor subunit levels in association with increased anxiety in the female rat. Brain Res. (2001) 910:55–66. doi: 10.1016/s0006-8993(01)02565-3 11489254 PMC4170586

[83] Bixo M Johansson M Timby E Michalski L Backstrom T. Effects of GABA active steroids in the female brain with a focus on the premenstrual dysphoric disorder. J Neuroendocrinol. (2018) 30:e12553. doi: 10.1111/jne.12553, PMID: 29072794

[84] Schiller CE Schmidt PJ Rubinow DR. Allopregnanolone as a mediator of affective switching in reproductive mood disorders. Psychopharmacology. (2014) 231:3557–67. doi: 10.1007/s00213-014-3599-x, PMID: 24846476 PMC4135022

[85] Lundgren P Strömberg J Bäckström T Wang M. Allopregnanolone-stimulated GABA-mediated chloride ion flux is inhibited by 3beta-hydroxy-5alpha-pregnan-20-one (isoallopregnanolone). Brain Res. (2003) 982:45–53. doi: 10.1016/S0006-8993(03)02939-1, PMID: 12915239

[86] Bäckström T Ekberg K Hirschberg AL Bixo M Epperson CN Briggs P. A randomized, double-blind study on efficacy and safety of sepranolone in premenstrual dysphoric disorder. Psychoneuroendocrinology. (2021) 133:105426. doi: 10.1016/j.psyneuen.2021.105426, PMID: 34597899

[87] Osborne LM Gispen F Sanyal A Yenokyan G Meilman S Payne JL. Lower allopregnanolone during pregnancy predicts postpartum depression: an exploratory study. Psychoneuroendocrinology. (2017) 79:116–21. doi: 10.1016/j.psyneuen.2017.02.012, PMID: 28278440 PMC5420429

[88] Leader LD O'Connell M VandenBerg A. Brexanolone for postpartum depression: clinical evidence and practical considerations. Pharmacotherapy. (2019) 39:1105–12. doi: 10.1002/phar.2331 31514247

[89] Fasipe OJ Agede OA Enikuomehin AC. Announcing the novel class of GABA-A receptor selective positive allosteric modulator antidepressants. Future Sci OA. (2020) 7:Fso654. doi: 10.2144/fsoa-2020-0108, PMID: 33437518 PMC7787135

[90] Dichtel LE Nyer M Dording C Fisher LB Cusin C Shapero BG. Effects of open-label, adjunctive Ganaxolone on persistent depression despite adequate antidepressant treatment in postmenopausal women: a pilot study. J Clin Psychiatry. (2020) 81:7602. doi: 10.4088/JCP.19m12887, PMID: 32558402 PMC7738196

[91] Althaus AL Ackley MA Belfort GM Gee SM Dai J Nguyen DP. Preclinical characterization of zuranolone (SAGE-217), a selective neuroactive steroid GABA(A) receptor positive allosteric modulator. Neuropharmacology. (2020) 181:108333. doi: 10.1016/j.neuropharm.2020.108333, PMID: 32976892 PMC8265595

[92] Hoffmann E Nomikos GG Kaul I Raines S Wald J Bullock A. SAGE-217, a novel GABA(A) receptor positive allosteric modulator: clinical pharmacology and tolerability in randomized phase I dose-finding studies. Clin Pharmacokinet. (2020) 59:111–20. doi: 10.1007/s40262-019-00801-0, PMID: 31338688 PMC6994455

[93] Carlini SV Deligiannidis KM. Evidence-based treatment of premenstrual dysphoric disorder: a concise review. J Clin Psychiatry. (2020) 81:6789. doi: 10.4088/JCP.19ac13071, PMID: 32023366 PMC7716347

[94] Maguire MJ Nevitt SJ. Treatments for seizures in catamenial (menstrual-related) epilepsy. Cochrane Database Syst Rev. (2019) 10:CD013225. doi: 10.1002/14651858.CD013225.pub2, PMID: 31608992 PMC6953347

[95] Rustichelli C Bellei E Bergamini S Monari E Baraldi C Castro FL. Serum levels of allopregnanolone, progesterone and testosterone in menstrually-related and postmenopausal migraine: a cross-sectional study. Cephalalgia. (2020) 40:1355–62. doi: 10.1177/0333102420937742, PMID: 32588652 PMC7575305

[96] Pinna G Almeida FB Davis JM. Allopregnanolone in postpartum depression. Front Glob Womens Health. (2022) 3:823616. doi: 10.3389/fgwh.2022.823616, PMID: 35558166 PMC9088875

[97] Yuan L Chen G Jia H Gao Y Liu L Li Y. Research status of GABA_A_ receptor. J Modern Clin Med. (2014) 40:89–92.

